# Global Population Genetic Analysis of *Aspergillus fumigatus*

**DOI:** 10.1128/mSphere.00019-17

**Published:** 2017-02-01

**Authors:** Eta Ebasi Ashu, Ferry Hagen, Anuradha Chowdhary, Jacques F. Meis, Jianping Xu

**Affiliations:** a Department of Biology, McMaster University, Hamilton, Ontario, Canada; b Department of Medical Microbiology and Infectious Diseases, Canisius Wilhelmina Hospital, Nijmegen, Netherlands; c Department of Medical Mycology, Vallabhbhai Patel Chest Institute, University of Delhi, Delhi, India; d Center of Expertise in Mycology, Radboud University Medical Center, Canisius Wilhelmina Hospital, Nijmegen, Netherlands; e Public Research Laboratory and Institute of Tropical Diseases Research, Hainan Medical University, Haikou, Hainan, China; Carnegie Mellon University

**Keywords:** *Aspergillus fumigatus*, ecological structure, gene flow, genetic populations, genetic recombination, geographic structure, microsatellite markers, triazole drug selection

## Abstract

*Aspergillus fumigatus* is a ubiquitous opportunistic fungal pathogen capable of causing invasive aspergillosis, a globally distributed disease with a mortality rate of up to 90% in high-risk populations. Effective control and prevention of this disease require a thorough understanding of its epidemiology. However, despite significant efforts, the global molecular epidemiology of *A. fumigatus* remains poorly understood. In this study, we analyzed 2,026 *A. fumigatus* isolates from 13 countries in four continents using nine highly polymorphic microsatellite markers. Genetic cluster analyses suggest that our global sample of *A. fumigatus* isolates belonged to eight genetic clusters, with seven of the eight clusters showing broad geographic distributions. We found common signatures of sexual recombination within individual genetic clusters and clear evidence of hybridization between several clusters. Limited but statistically significant genetic differentiations were found among geographic and ecological populations. However, there was abundant evidence for gene flow at the local, regional, and global scales. Interestingly, the triazole-susceptible and triazole-resistant populations showed different population structures, consistent with antifungal drug pressure playing a significant role in local adaptation. Our results suggest that global populations of *A. fumigatus* are shaped by historical differentiation, contemporary gene flow, sexual reproduction, and the localized antifungal drug selection that is driving clonal expansion of genotypes resistant to multiple triazole drugs.

**IMPORTANCE** The genetic diversity and geographic structure of the human fungal pathogen *A. fumigatus* have been the subject of many studies. However, most previous studies had relatively limited sample ranges and sizes and/or used genetic markers with low-level polymorphisms. In this paper, we characterize a global collection of strains of *A. fumigatus* using a panel of 9 highly polymorphic microsatellite markers. Using these markers, we analyze 2,026 isolates, which is ~3 times the number of isolates reported so far in previous studies. Our analyses suggest that *A. fumigatus* contains historically differentiated genetic populations but that its evolution is significantly impacted by contemporary forces such as widespread gene flow and local antifungal drug pressure. In the wake of a global rise in resistance to azoles in fungal pathogens, our findings should aid in developing management strategies to mitigate current increases to azole resistance.

## Abstract (precis)

The genetic diversity and geographic structure of the human fungal pathogen *A. fumigatus* have been the subject of many studies. However, most previous studies had relatively limited sample ranges and sizes and/or used genetic markers with low-level polymorphisms. In this paper, we characterize a global collection of strains of *A. fumigatus* using a panel of 9 highly polymorphic microsatellite markers. Using these markers, we analyze 2,026 isolates, which is ~3 times the number of isolates reported so far in previous studies. Our analyses suggest that *A. fumigatus* contains historically differentiated genetic populations but that its evolution is significantly impacted by contemporary forces such as widespread gene flow and local antifungal drug pressure. In the wake of a global rise in resistance to azoles in fungal pathogens, our findings should aid in developing management strategies to mitigate current increases to azole resistance.

## INTRODUCTION

*Aspergillus fumigatus* is a globally distributed opportunistic human fungal pathogen. While its primary ecological niche is decomposing organic matter, *A. fumigatus* is ubiquitous in the environment and can be found in a broad range of ecological niches. In humans, it can colonize the lung and several other body sites, causing infections collectively known as aspergillosis. *A. fumigatus* is a major source of morbidity and mortality in immunocompromised patients causing approximately 4 to 5 million cases of allergic bronchopulmonary aspergillosis globally, approximately 10% of which become chronic (1). Invasive aspergillosis is the most severe form of aspergillosis; it is estimated to have a global annual incidence of up to 10% and a mortality rate as high as 90% in high-risk groups (2, 3).

Compounding the increasing incidences of aspergillosis is antifungal drug resistance associated with such infections. Targeting the highly conserved fungal ergosterol biosynthesis pathway, triazoles are the most common and effective drugs used in first-line treatment of aspergillosis. However, due to the increasing frequency of azole resistance in *A. fumigatus*, treatment failures are increasingly common and drug-resistant aspergillosis has become a significant global health issue (4, 5). The medical significance of aspergillosis and azole resistance has attracted significant attention from microbiologists, health care workers, and public health agencies worldwide. Indeed, several studies have examined the genetic relationships among *A. fumigatus* strains from different geographic areas (6–10). For instance, Debeaupuis et al*.* (6) examined 879 isolates from five countries using restriction fragment length polymorphisms (RFLP) based on the Southern hybridization pattern of a retrotransposon-like element and reported no evidence of genetic differentiation between environmental and clinical isolates. Similarly, Rydholm et al*.* (7) examined patterns of genetic variation at three intergenic loci for 70 isolates from 22 countries using multilocus sequence typing (MLST) and obtained similar results. Interestingly, Pringle et al. (8) examined 63 isolates collected from 14 countries based on DNA sequence data at five loci and identified two globally distributed and genetically differentiated clusters. More recently, in 2009, another study analyzed 55 isolates from four countries using amplified fragment length polymorphisms (AFLP) and showed some evidence of differentiation by geographic and ecological origins (9). Klaassen et al. (2012) analyzed 255 isolates from the Netherlands using 20 molecular markers, including nine highly polymorphic microsatellite markers, and found no evidence of differentiation among geographic populations within the Netherlands (10). However, they found that samples from the Netherlands could be grouped into five genetic populations and that all the isolates containing the multiple-triazole-resistant allele at the *CYP51A* gene belonged to only one of the five genetic populations (10). Discrepancies in results obtained by these studies were likely due to the use of relatively small sample sizes and/or different markers. It was previously suggested that using markers with higher discriminatory power and analyzing more-diverse and larger sample sizes would likely produce more-robust and more-consistent results for *A. fumigatus* (10).

Over the last few decades, several molecular methods, including MLST, microsatellite markers, randomly amplified polymorphic DNA (RAPD) typing, PCR-RFLP, RFLP detected through Southern hybridization, and AFLP, have been used for genotyping *A. fumigatus* (11, 12). However, results obtained with some of these typing methods, such as RAPD and AFLP, have limited reproducibility and are difficult to interpret or compare among laboratories. Among these methods, the use of microsatellite markers has emerged as the best typing method in terms of reproducibility, costs, and discriminatory power (13). For example, Klaassen et al. showed that of the 225 *A. fumigatus* genotypes revealed by 20 combined markers—9 microsatellite, 1 indel, and 10 sequence/PCR-typing markers—224 could be recognized by the 9 microsatellites alone (10). Although the nine microsatellite markers in this set are likely neutral and suitable for population genetic and epidemiological studies, they have not been used to characterize the global population of *A. fumigatus* (10, 14).

Using the aforementioned nine microsatellite markers, we analyzed 2,026 *A. fumigatus* isolates from 13 countries in 4 continents. We aimed to (i) critically examine the genetic relationships of the isolates within and among geographic populations of *A. fumigatus*, (ii) investigate how antifungal drug susceptibility patterns are related to genetic variations, and (iii) evaluate the roles of sexual and asexual reproduction in shaping the evolution of azole resistance globally. We tested the Baas Becking hypothesis that “everything is everywhere, the environment selects” (15). Due to its close associations with humans and human activities, both intrinsic natural factors and anthropogenic factors are expected to influence the genetic structure of *A. fumigatus* populations. For example, diverse microbial populations can often be structured by environmental factors (16–20), including the use of antifungal drugs. Thus, we hypothesize that there should be some genetic differentiation among samples of *A. fumigatus* with regard to geographic origin, triazole resistance, and other ecological factors. Indeed, recent investigations identified that local clonal expansion played a significant role in the spread of triazole resistance genotypes in the Netherlands and India (10, 21–23). However, given the abundance of asexual spores in *A. fumigatus*, their capability of long-distance dispersal, and their nonfastidious requirements for growth and reproduction, gene flow was expected to be common among geographic populations of *A. fumigatus*, potentially obscuring the geographic and ecological patterns of genetic variations. Below, we describe the relative roles of various factors that impact the global *A. fumigatus* population structure.

## RESULTS

### Limited but statistically significant geographic and ecological niche contributions to genetic variation.

Analysis of molecular variance (AMOVA) showed that 6% of the total genetic variation was contributed by geographic separation, with the majority of genetic variation found within individual geographic populations (*P* = 0.001) (see Fig. S1 in the supplemental material). Several pairwise geographic differentiations were significant, with the biggest differentiations found between India and other countries, including those between India and China (PhiPT [*p*airwise population *h*eterogeneity *i*ndex of the *p*roportion of *t*otal genetic variance] = 0.375, *P* = 0.001), India and Italy (PhiPT = 0.375, *P* = 0.001), and India and Australia (PhiPT = 0.357, *P* = 0.001). Similarly, we observed relatively limited but statistically significant genetic differentiations based on ecological niches. Among the 2,026 isolates, 434 had unspecified ecological origins and they were excluded from this analysis. The remaining 1,592 isolates were classified into four ecological niches (clinical, air, water, and soil), and only 3% of the total genetic variation was contributed by such ecological niche separation (*P* = 0.001) (see Fig. S2).

10.1128/mSphere.00019-17.1 FIG S1  Contribution of geographic separation to genetic variation in the global sample of 2,026 *Aspergillus fumigatus* isolates. Pie charts show percentages of variations among and within populations that can be explained by grouping isolates by geographic origin. PhiPT values are in the lower diagonal of the pairwise comparison table. Probabilities based on 999 permutations are shown above the diagonal. Download FIG S1, PDF file, 0.2 MB. Copyright © 2017 Ashu et al. 2017 Ashu et al. This content is distributed under the terms of the Creative Commons Attribution 4.0 International license.

10.1128/mSphere.00019-17.2 FIG S2  Contribution of ecological niches to the total genetic variation. A total of 1,592 isolates from our data set had known ecological (environmental or clinical) origins. Pie charts show percentages of variations among and within populations that can be explained by grouping isolates by ecological origin. PhiPT values are in the lower diagonal of the pairwise comparison table. Probabilities based on 999 permutations are shown above the diagonal. Download FIG S2, PDF file, 0.2 MB. Copyright © 2017 Ashu et al. 2017 Ashu et al. This content is distributed under the terms of the Creative Commons Attribution 4.0 International license.

### Distinct genetic clusters and evidence of historical differentiation.

**FIG 1:**
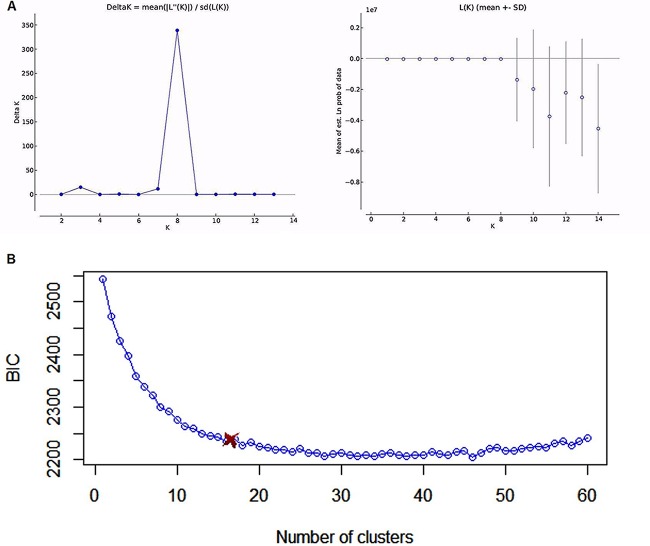
The optimal number of genetic clusters inferred by STRUCTURE and DAPC for our data set. (A) Rate of change in the log probability (prob.) of data between successive runs of K (ΔK) (52) and the average posterior probability (ln K) for each K (i.e., K 1 to 14). The optimal predicted number of populations (K) for our set of isolates is eight. est., estimated. (B) Plot of the optimal number of clusters (K) versus the Bayesian information criterion (BIC). The BIC rate of change drops considerably after 8 clusters and flattens after ~14 clusters.

Given the diverse geographic and ecological niches of the samples, the relatively limited contributions of geography and ecological niche to the total genetic variations were surprising. Among the factors contributing to the limited geographic and ecological differentiation were the high levels of allelic and genotypic diversities within most geographic and ecological niche populations (see Fig. S3). In total, 1,230 multilocus microsatellite genotypes were found among the 2,026 analyzed isolates. In order to examine potentially divergent genetic clusters, the Bayesian algorithm as implemented by STRUCTURE software was used. However, since STRUCTURE was unable to analyze all 2,026 isolates at the same time to infer an optimal number of genetic clusters from our set of isolates, a clone-corrected sample of 1,230 genotypes was used instead. Here, one randomly selected isolate was picked to represent each individual multilocus genotype. Structure analyses separated the 1,230 genotypes into eight genetic cluster populations, Pop 1 to Pop 8 (Fig. 1). Less than 1% of all 1,230 genotypes were assigned to Pop 1 (8/1,230), whereas 18.7% (230/1,230), 7.7% (95/1,230), 13.4% (165/1,230), 16.7% (206/1,230), 7.3% (90/1,230), 9.8% (120/1,230), and 25.7% (316/1,230) were assigned to Pop 2, 3, 4, 5, 6, 7, and 8, respectively.

10.1128/mSphere.00019-17.3 FIG S3  Mean allelic information patterns across geographical and genetic populations. Na, number of different alleles; Ne, number of effective alleles [1/(Sum pi^2^)]; I, Shannon’s information index {−1 × Sum [pi × Ln(pi)]}; h, diversity [1 − Sum pi^2^]; uh, unbiased diversity {[*N*/(*N* − 1)] × h}, where pi is the frequency of the *i*th allele for the population and Sum pi^2^ is the sum of the squared population allele frequencies. Download FIG S3, PDF file, 0.3 MB. Copyright © 2017 Ashu et al. 2017 Ashu et al. This content is distributed under the terms of the Creative Commons Attribution 4.0 International license.

AMOVA performed on the eight STRUCTURE-inferred genetic clusters showed that 18% of the total genetic variance was found among the eight clusters (*P* = 0.001) (Fig. S4). One of the eight clusters (Pop 1) was found in only one country, Belgium, suggesting that Belgium was most likely the geographic origin for this cluster. However, all other clusters contained isolates from at least 9 of the 13 countries analyzed here (Table 1). The inference of the eight genetic clusters on the basis of the STRUCTURE analyses is supported by results from the discriminant analysis of principal components (DAPC), a non-model-based approach implemented in R. We note that, although the Bayesian information criterion (BIC) value was still decreasing after separation of the genotypes into eight genetic populations, the rate of change dropped considerably after that point (Fig. 1). The existence of distinct genetic clusters is consistent with historical differentiation within *A. fumigatus*.

10.1128/mSphere.00019-17.4 FIG S4  Population differentiation among STRUCTURE-inferred genetic clusters. Our clonally corrected data set of 1,230 genotypes was used to infer the number of genetic cluster. Pie charts show percentages of variations among and within populations. PhiPT values are in the lower diagonal of the pairwise comparison table. Probabilities based on 999 permutations are shown above the diagonal. Download FIG S4, PDF file, 0.2 MB. Copyright © 2017 Ashu et al. 2017 Ashu et al. This content is distributed under the terms of the Creative Commons Attribution 4.0 International license.

**TABLE 1:** Global distribution of the 8 inferred genetic clusters^a^

Genetic clusters (no. of genotypes)	% prevalence (no. of genotypes) in indicated country												
	Australia	Belgium	China	Cuba	France	Germany	India	Italy	Netherlands	Norway	Spain	Switzerland	United States
Pop 1 (8)		100 (8)											
Pop 2 (230)		4 (9)	2 (5)	2 (4)	1 (3)	5 (12)	4 (10)		43 (99)	8 (18)	10 (24)	6 (14)	14 (32)
Pop 3 (97)		7 (7)	1 (1)	1 (1)	7 (7)	2 (2)	1 (1)		66 (66)	4 (4)	2 (2)	8 (8)	
Pop 4 (165)	1 (2)	5 (9)		1 (1)	5 (9)	7 (11)	1 (1)	1 (2)	61 (101)	8 (13)	5 (8)	2 (3)	3 (5)
Pop 5 (206)	1 (2)	6 (12)			3 (7)	2 (5)	2 (4)		50 (102)	8 (17)	14 (29)	8 (16)	6 (12)
Pop 6 (90)		6 (5)			3 (3)	19 (17)	3 (3)		47 (42)	7 (6)	9 (8)	6 (5)	1 (1)
Pop 7 (120)	1 (1)	3 (4)				3 (3)	1 (1)		41 (49)	44 (53)	3 (4)	3 (4)	1 (1)
Pop 8 (316)		6 (19)	1 (2)	1 (4)	6 (20)	1 (4)	2 (5)	1 (2)	56 (178)	13 (42)	7 (21)	4 (12)	2 (7)

a Numbers in parentheses represent the numbers of genotypes in individual genetic clusters and in geographic subpopulations of said genetic clusters.

While STRUCTURE analysis is based on the use of allelic association patterns to separate reproductively isolated strains into genetic populations/clusters and can be used to indicate historical differentiations, other indicators can also be used to support the hypothesis of historical differentiation in *A. fumigatus*. In this study, six of the eight clusters had at least five private alleles each at the nine analyzed loci. The percentages of private alleles by genetic cluster were as follows: for Pop 2, 23% (53/230); for Pop 3, 5.3% (5/95); for Pop 4, 15.8% (26/165); for Pop 5, 12.6% (26/237); for Pop 6, 25.6% (23/237); and for Pop 8, 23.9% (104/237). Pop 1 and Pop 7 had no private alleles.

### Evidence for recombination within genetic and geographic populations and hybridization among genetic clusters.

We found various levels of linkage equilibrium and evidence of recombination within each of the eight *A. fumigatus* genetic clusters (Table 2). For example, all eight genetic clusters had phylogenetically incompatible pairs of loci (Table 2). However, as expected, evidence for clonal reproduction was also found and the findings led to a strong rejection of the null hypothesis of random recombination for all eight genetic clusters (*P* < 0.01). Similarly, evidence for both clonality and recombination was also found within individual geographic populations of *A. fumigatus* (Table 2). Specifically, phylogenetic incompatibility was found in each of the 13 geographic populations. We further tested the effects of clone correction on the index of association, and our analyses showed that the clonally corrected geographic samples all had reduced levels of linkage disequilibrium (LD) but still deviated from random recombination. Populations from four countries (Australia, China, Cuba, and Italy) were each represented by less than 10 isolates after clonal correction, and these populations were excluded from this analysis.

**TABLE 2:** Indices of association and phylogenetic compatibility within individual genetic clusters and geographic populations^a^

Population	Index of association	Phylogenetic compatibility
Pop 1	1.18	0.97
Pop 2	0.30	0.00
Pop 3	0.38	0.06
Pop 4	0.67	0.00
Pop 5	0.78	0.00
Pop 6	0.40	0.20
Pop 7	0.37	0.06
Pop 8	0.34	0.00
Belgium	2.24 (0.73)	0.00
France	1.72 (0.95)	0.00
Germany	1.38 (0.88)	0.00
India	6.66 (0.30)	0.30
Netherlands	0.75 (0.69)	0.00
Norway	0.89 (0.80)	0.00
Spain	1.72 (0.48)	0.00
Switzerland	0.74 (0.55)	0.00
United States	0.88 (0.84)	0.00

a The eight genetic clusters are represented by Pop 1 to Pop 8.

Some of the phylogenetic incompatibilities observed within individual geographic populations may represent evidence of hybridization and genetic recombination between different genetic clusters. Specifically, a number of microsatellite genotypes within the eight genetic populations showed evidence of ancestry corresponding to two or more genetic clusters. For example, certain strains within Pop 1 had mixtures of alleles from Pop 2, 3, 4, and 8 in Belgium. Specifically, loci 2A and 2B of Pop 1 had allelic matches with those in Pop 2; locus 2C had an allelic match with that in Pop 4; loci 3A and 3B had allelic matches with that in Pop 3; locus 4A had an allelic match in Pop 2 and 4; and locus 4C had a match in Pop 8. Locus 4B did not have any exact matches with other genetic clusters within Belgium and was significantly differentiated from all other genetic clusters in Belgium. While these results are consistent with potential recent hybrid origins of certain strains in Pop 1, there is another possibility: the sharing of alleles among genetic clusters might represent the results of recent mutational convergence from different populations. Furthermore, interestingly, for some strains with evidence of multiple ancestries, the putative ancestral populations were often closely related. Thus, for these strains, there is a third possibility, i.e., that their mixed ancestries were due to incomplete lineage sorting among the ancestral polymorphisms.

### Evidence of contemporary gene flow.

Our results showed abundant evidence of gene flow among ecological and geographic populations of *A. fumigatus* (see Fig. S1 and S2). For example, of the total of 625 environmental (air, soil, and water) and 582 clinical *A. fumigatus* genotypes, 90 were shared between the two ecological types (see Fig. S5). However, the numbers of genotypes shared between clinical and environmental samples differed from country to country, with some countries having as many as 50 genotypes shared between the clinical and environmental sources.

10.1128/mSphere.00019-17.5 FIG S5  Genotype matches between environmental and clinical populations. Allelic profiles of all 90 genotypes with nine matches of microsatellite loci between the two ecological populations are shown. The population labeled “Env” had an environmental origin; however, its specific (air, water, or soil) environmental niche was unknown. Download FIG S5, PDF file, 0.3 MB. Copyright © 2017 Ashu et al. 2017 Ashu et al. This content is distributed under the terms of the Creative Commons Attribution 4.0 International license.

**FIG 2:**
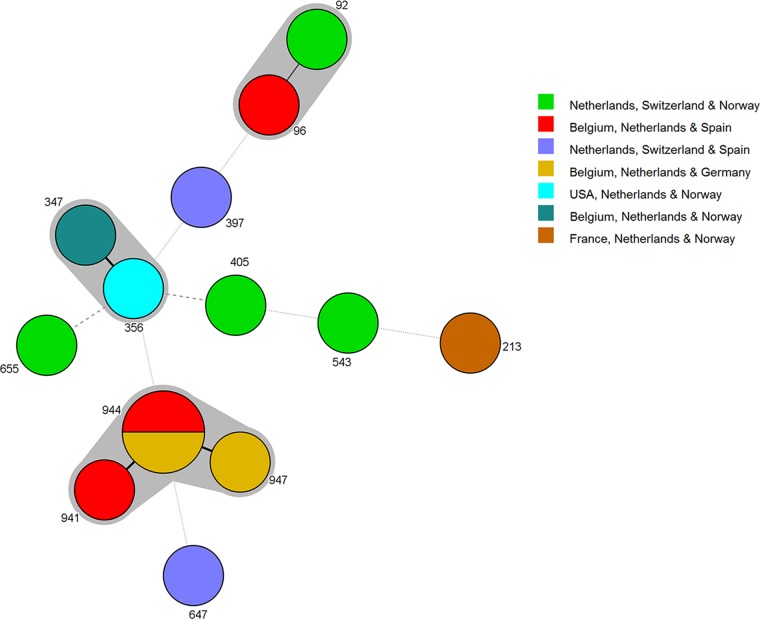
Minimum spanning tree of all genotypes identified in at least three countries. Each circle represents a genotype. Thick, short, solid lines connect variants that differ by alleles at one of the nine loci; thick, longer, solid lines connect variants with different alleles at two loci; dashed and dotted lines connect variants at four and more loci, respectively. Unique colors were assigned to represent the countries in which genotypes were identified. The gray shading depicts genotypes belonging to the same clonal complex.

Similarly, there is abundant evidence for gene flow among geographic populations. First, pairwise PhiPT values between most geographic populations were low (Fig. S1). Second, the allelic distribution patterns across geographic populations revealed that many alleles were shared by countries located far from each other (Fig. S3). Third, certain genotypes were shared by isolates from countries separated by long distances. For example, genotype 115 has a microsatellite allelic combination of 13-10-9-10-11-9-8-9-19 at loci 2A-2B-2C-3A-3B-3C-4A-4B-4C, respectively, with the allelic numbers representing the number of di- or trinucleotide repeats at each of the microsatellite loci. This genotype was isolated from an air sample collected in Belgium and from a patient in the United States, while genotype 1153 with the allelic combination 25-19-19-26-19-17-10-16-8 was isolated from an air sample collected in Norway and from a patient in India. Genotype 356 with the allelic combination 18-12-8-28-10-20-9-9-5 was isolated from both an air sample and a patient in the Netherlands and was also isolated from a patient in the United States. Furthermore, cluster analysis performed using the minimum spanning network identified evidence of dispersal for clonal complexes across countries that are up to 1,800 km apart (Fig. 2). Indeed, the Mantel test revealed no significant correlation between genetic distance and geographic distance (*r*^2^ = 0.002) at the global scale.

While the allele and genotype sharing between geographically distant countries such as those described above represents long-distance gene flow, evidence for gene flow among regions within individual countries is also abundant. For example, the samples from within India were obtained from diverse locations separated by hundreds of kilometers and several genotypes were found to be widely distributed across several locations. Taken together, our results suggest frequent gene flows among regional and continental populations of *A. fumigatus*.

### Evidence of local contemporary drug selection followed by clonal expansion.

About 6% of the isolates in our data set showed resistance to triazole antifungal drugs. Overall, there was a small but statistically significant genetic difference between the triazole-susceptible and triazole-resistant samples (PhiPT = 0.063, *P* = 0.001). Interestingly, while the triazole-susceptible populations showed relatively little genetic difference among geographic populations, the triazole-resistant isolates separated based on geographic origins showed a large amount of genetic differentiation (PhiPT = 0.57, *P* = 0.001) (see Fig. S6). We further tested the hypothesis that the differences between the triazole-susceptible and triazole-resistant samples were due to local clonal expansion. Indeed, after clonal correction, the level of differentiation among geographic populations of the triazole-resistant samples was reduced significantly (PhiPT = 0.23, *P* = 0.001). Taken together, the results from these analyses suggest that clonal expansion of triazole resistance genotypes plays a significant role in geographic structuring of *A. fumigatus*.

10.1128/mSphere.00019-17.6 FIG S6  Contribution of geographic separation to genetic variation in the triazole-resistant sample of 102 isolates. Pie charts show percentages of variations among and within populations that can be explained by grouping resistant isolates by geographic origin. A total of 106 resistant isolates from five countries were identified; however, countries with fewer than seven isolates were excluded from our analysis of molecular variance. PhiPT values are in the lower diagonal of the pairwise comparison table. Probabilities based on 999 permutations are shown above the diagonal. Download FIG S6, PDF file, 0.2 MB. Copyright © 2017 Ashu et al. 2017 Ashu et al. This content is distributed under the terms of the Creative Commons Attribution 4.0 International license.

## DISCUSSION

We investigated the global population structure using nine highly informative microsatellite markers to analyze a comprehensive set of isolates from four continents. Our analyses showed the existence of eight significantly differentiated genetic clusters within the global sample of* A. fumigatus*, a result consistent with historical differentiation. However, we noted low differentiation levels and frequent sharing of alleles and genotypes among geographic and ecological populations, suggesting that contemporary gene flow is prevalent. Our analyses also identified abundant signatures of both sexual recombination and local clonal expansion. Local clonal expansions were most evident in geographic populations where resistance to triazole antifungal drugs was prevalent. Taken together, our results suggest that both historical and contemporary factors have played a significant role in shaping the global population structure of *A. fumigatus*.

Structure analyses separated all 1,230 genotypes into 8 genetic clusters, 3 to 6 more than previously identified (8, 10). In 2005, Pringle and colleagues reported the presence of two globally distributed *A. fumigatus* genetic clusters and suggested that one of these clusters could be a cryptic *Aspergillus* species. In 2012, Klaassen et al. (10) identified five genetic clusters in the Netherlands based on analyses of 255 isolates representing 225 multilocus genotypes. In our study, there were 1,081 isolates representing 637 multilocus genotypes from the Netherlands, revealing two additional genetic clusters in that country (now seven genetic populations total in the Netherlands; Table 1). The identification of additional clusters over those identified in previous studies suggests that there are likely more genetic clusters in both the analyzed geographic regions and those not analyzed in this study. While the study by Klaassen et al. (10) and our current study used the same genetic markers and the same assumptions in inferring genetic clusters, we should note that our current inferred “optimal cluster number” (K) of 8 represents only the samples we have and is based on a series of assumptions that might not be entirely valid in nature (24). Indeed, the multivariate clustering approach implied a less clear-cut solution (Fig. 1). However, there is some consistency between the results obtained from the multivariate and the Bayesian clustering methods. Interestingly, most genetic clusters inferred by both methods were globally distributed, and the majority of triazole-resistant genotypes clustered into a single genetic population.

Comparably to results published previously, our analyses revealed that geographic populations often contain strains of divergent genotypes from different genetic clusters. At present, the putative geographic origin(s) for each of the eight genetic clusters is unresolved. Generally, the geographic center of origin should contain the highest allelic and genotypic diversities. In fungi, abundant evidence of sexual recombination has also been used as a signature for ancestral populations. However, uneven sample sizes and incomplete sampling in many countries make any conclusions at present extremely tentative (Table 1). Additional sequence data from multiple gene loci as well as more samples could help resolve this issue.

Though evidence for both local and long-distance dispersals was prevalent in our samples of *A. fumigatus*, limited but statistically significant population differentiations were observed among certain geographical or ecological populations. Finlay argued that microbes smaller than about 2 µm in size are unlikely to be restricted by geographical barriers in their dispersals (25). However, it is not uncommon to see geographical differentiation in microbial eukaryotes (26, 27). For example, using DNA sequence variation in four protein-coding genes, Kasuga et al. showed population differentiation by geographic origin in the human-pathogenic fungus *Histoplasma capsulatum* (26). In contrast, the global population structure of the wheat fungal pathogen *Mycosphaerella graminicola* is characterized by frequent gene flow (28). In *A. fumigatus*, the low but statistically significant levels of differentiation were likely due to historical separations; however, these genetic differentiations are being broken down by contemporary gene flow.

Our evidence for gene flow among regional populations at the global scale extends observations reported earlier based on smaller sample sizes and/or geographically limited samples (6–10). Gene flow within and between geographic populations of *A. fumigatus* can be accomplished by wind-aided spore dispersal or by anthropogenic activities. *A. fumigatus* can reproduce both sexually and asexually and can produce abundant ascospores and conidiospores, respectively, in the process. These hydrophobic spores can become readily airborne and disperse by wind. The rate of emission of *A. fumigatus* conidia from an undisturbed compost pile is estimated to be 8 × 10^3^ to 11 × 10^3^ CFU/m^2^/s at the mean wind speed of 1 m/s (29). Our data suggested two major dispersal patterns. The first and most frequently observed dispersal pattern involved intermediate-distance dispersals (IDD) and short-distance dispersals (SDD). For example, it was common to find several isolates from diverse locations and ecological niches within the same country sharing the same genotype. Similarly, despite relatively limited genotype sharing, allele frequencies among local populations within the Netherlands were very similar (10). Frequent gene flow can mask population differentiation and represent a major challenge for molecular epidemiologists in their efforts to track and contain fungal strains of public health interest.

The second dispersal pattern is long-distance dispersal (LDD), which can be assisted or not assisted by travel by humans. Assisted LDD refers to dispersal involving humans or associated with human artifacts whereby travelers can carry conidia on/in their bodies or on infested inanimate objects from one region to another far away. In contrast, unassisted LDD involves dispersals without human interventions, e.g., by air currents. Our data here show that certain microsatellite genotypes are found in countries up to 7,500 km apart. For example, genotype 115 was isolated from air in Belgium and from patients in the United States. However, whether dispersal of this genotype and other shared genotypes of *A. fumigatus* was assisted or not assisted by humans is currently unknown. Documenting and understanding the frequency of and mechanism(s) for such long-distance dispersals are of utmost importance in containment and eradication of pathogens. LDD events have been previously reported to play a crucial role in fungal pathogen recolonization, invasion, and adaptation to host resistance (30). For example, it was suggested that cyclonic winds introduced *Puccinia melanocephala* uredospores into the Dominican Republic from Cameroon, in early June of 1978 (31). Similarly, *Cryptococcus deuterogattii* (previously *C. gattii* AFLP6/VGII) was likely spread from Vancouver Island to mainland British Columbia, Canada, and the Pacific Northwest of the United States in dust on cars and under shoes of travelers that took the ferry to Vancouver City (32). Owing to the fact that *A. fumigatus* is ubiquitous, LDD likely plays a limited role in future pathogen invasion or recolonization. However, in view of adaptation to host resistance and recent increases in azole resistance, our finding of single-event long-distance dispersal would be of great significance to epidemiologists, although all cases of LDD identified in this study so far involved only triazole-susceptible isolates.

Gene flow can have both advantageous and disadvantageous consequences with respect to the selective maintenance of genetic variation within and between microbial populations. For instance, gene flow is thought to limit population divergence and hence local selection. Gene swamping into naive populations favors fixing of alleles with the best average reproductive success, thereby counteracting the stability of local selection (33). However, the concept of gene flow moderating population divergence has been contested by observations indicating that several species, including fungal species, show little genetic differentiation even though they lack sufficient gene flow to counteract divergence (34). On the other hand, gene flow is thought to facilitate adaptation by disseminating beneficial alleles. However, dissemination of highly beneficial alleles does not necessarily require high levels of gene flow (34). In *A. fumigatus*, frequent gene flow, which is known to facilitate dissemination of alleles highly beneficial to the fungus such as those conferring triazole resistance, can be attributed to the abundance of asexual spores, their capability of long-distance dispersal, and their nonfastidious requirements for growth and reproduction. The limited geographic differentiation, the presence of multiple private alleles within individual geographic and genetic populations, and the evidence for gene flow among global populations of *A. fumigatus* indicate that the evolution of *A. fumigatus* will be continuously impacted by both local and global factors.

Our analyses suggest that genetic Pop 2 and Pop 8 are the most widely distributed and are recombining. They also contained the most private alleles. The previous study by Klaassen et al. (10) revealed that all multitriazole-resistant strains with the TR34/L98H mutations at the *CYP51A* gene from the Netherlands belonged to one genetic cluster (Pop 3 in their study and Pop 8 here in our study). Interestingly, while Pop 8 contained 80% (35/44) of all triazole-resistant genotypes, triazole resistance genotypes were also found in other genetic clusters. However, our results do indicate that the frequency of triazole resistance in Pop 8 (11%, 35/316) was at least four times higher than that in any other genetic population, suggesting that special attention should be paid to understanding this genetic cluster. Since the gene(s) related to resistance is not known to be tightly linked to the analyzed nine microstatellite loci, the tight clustering of most triazole-resistant isolates in Pop 8 suggests three possibilities. The first possibility is that Pop 8 is more frequently distributed in geographic regions where triazole drug use is very common. It has been previously hypothesized that extensive agricultural use of azole fungicides in *A. fumigatus* in India and elsewhere leads to resistance (2, 35, 36). Although azoles are used worldwide, the absolute amounts used differ among countries (37). The differences in the types and absolute amounts of azole fungicides used by individual regions and countries could act as distinct selective pressures to generate the different frequencies of triazole-resistant strains. This hypothesis was supported by evidence indicating that resistant isolates grouped by geographic origin were genetically highly differentiated (*P* = 0.001). Using *Escherichia coli* as a model organism, it has been experimentally demonstrated that the strength of antibiotic exposure plays a significant role in the evolution of antibiotic resistance (38). The second possibility is that, when exposed to triazole drugs, strains in this genetic cluster are more likely than those in other genetic clusters to develop triazole resistance. If so, then caution should be taken in using triazole drugs when treating infections caused by strains in this genetic cluster. Similar clade-specific patterns have also been shown in the plant-pathogenic fungi *Puccinia graminis* f. sp. *tritici* and *Pyrenophora tritici-repentis* (39, 40). The third possibility is that strains in Pop 8 are more receptive than those in other clusters in accepting triazole-resistant genes via mating and recombination. Indeed, even though the early study by Klaassen et al. (10) did not show evidence of recombination for this cluster in the Netherlands, our analysis of the expanded samples in Pop 8 showed the most evidence of recombination in our samples where all pairs of loci among the nine microsatellite markers showed phylogenetic incompatibility. The occurrence of recombination within and between *A. fumigatus* genetic populations could have significant implications in the initiation and dissemination of resistant/virulent strains capable of causing aspergillosis outbreaks (41).

Although sexual reproduction can generate genotypic variation among *A. fumigatus* populations which may allow faster adaptation to host resistance, it can, however, be costly in terms of energy and time and may have no measurable advantage in new ecological niches (42). This could possibly explain why the other genetic populations (Pop 1 and Pop 3 to 7) evolved predominantly clonally and contain significantly fewer resistant isolates. Furthermore, although most triazole-resistant genotypes belonged to a highly recombinant genetic cluster, our data, as well as data from previous studies, showed that the expansion of triazole-resistant strains at the local (country) level is predominantly clonal (10, 21–23). Such localized clonal expansion is a significant factor that shapes the current *A. fumigatus* population structure. Indeed, the clonal spread of adaptive recombinant progeny resistant to multiple triazole drugs in India was the main cause of the significant genetic differentiations between the Indian *A. fumigatus* population and those isolated elsewhere in the world.

In conclusion, using a large number of isolates from geographically and ecologically diverse regions, our study allowed us to address several fundamental questions about the global population of *A. fumigatus*. Our findings go beyond those previously identified by other studies. We report limited but statistically significant genetic differentiations among geographic and ecological populations of *A. fumigatus*. The identification of eight genetically differentiated clusters is consistent with historical differentiation, but contemporary gene flows are blurring the historical patterns. Interestingly, unlike the triazole-susceptible samples, where geographic populations were largely undifferentiated, triazole-resistant samples were significantly differentiated according to geographic region. The differences in triazole usage among the countries have likely contributed to the genetic differences among the triazole-resistant samples. Though evidence for clonality was found in all geographic, ecological, and genetic populations, we also found evidence for recombination in all analyzed populations, a result different from those reported in earlier studies (6–10). This difference is especially noteworthy for Pop 8, the dominant genetic cluster containing most of the triazole-resistant strains, where no evidence of recombination was found in an earlier study (10) but abundant evidence for recombination was found in our current study. However, despite the large sample size and broad geographic and ecological representations in our samples, additional genetic diversities and other types of genetic relationships among samples could exist when additional samples are analyzed. Our results provide important data for future assessment of *A. fumigatus* migration patterns. From a practical perspective, our findings should aid in better tracking and management of aspergillosis outbreaks.

## MATERIALS AND METHODS

### Isolates used for analyses and genotyping.

Samples used in this study were obtained as part of collaborative studies between Canisius Wilhelmina Hospital and research centers in several countries (4, 10, 14, 43–49). A total of 2,026 isolates from 13 countries in 4 continents were genotyped and included in our analyses. Genotyping was performed with a panel of nine short tandem repeats (STRAf 2A, 2B, 2C, 3A, 3B, 3C, 4A, 4B, and 4C) as previously described (14).

### Identification of genetic clusters.

In order to investigate the existence of distinct genetic clusters, we used both multivariate and model-based Bayesian clustering as implemented in the ADEGENET package in R version 3.0 and STRUCTURE software version 2.3 (50, 51). Multivariate clustering was used to complement Bayesian clustering, bearing in mind that this approach assumes linkage equilibrium within clusters (50). However, multivariate clustering cannot be solely used for clustering analysis as its efficiency is limited by correlations between variables (alleles). Moreover, the contravention of the assumption of uncorrelated alleles is further emphasized in the presence of linkage disequilibrium. When multivariate clustering was used, the optimal number of clusters (K) was inferred based on BIC (50). For model-based Bayesian clustering, we chose the admixture and the correlated allele frequencies between population options as our ancestry and frequency models, respectively. Markov chain Monte Carlo (MCMC) simulations were run for K = 1 to 14. However, given the huge data set, the MCMC sampling scheme was run for only 9,000 iterations with a burn-in period of 1,000. Notwithstanding, summary statistics values (Alpha, Fst, and likelihood) seemed to have converged at 10^4^ iterations, thereby validating the run length. Two approaches were used to identify the optimal number of clusters. The *ad hoc* statistic DK, based on the rate of change in the log probability of data between successive runs of K, was calculated as previously recommended (52). The second approach recommended by Pritchard et al. (2000) calculates the average log probability [LnP (D)] of each K value (51).

### Differentiations among samples separated by geographic, ecological, and triazole susceptibility patterns.

In order to identify the potential contributors to the observed genetic variation, the pairwise samples grouped based on different criteria were compared using the GenAlEx version 6.5 (53). In these analyses, three criteria were used to group the samples: their triazole susceptibility status (triazole susceptible versus triazole resistant), their ecological niche (clinical, soil, air, or aquatic), and their geographical origin (country). Cluster analysis was done using the minimum spanning tree algorithm as implemented by Bionumerics version 7.0 (Applied Maths, Saint-Martens-Latem, Belgium).

### Analyses of genetic variability, allelic diversity, and recombination.

Nei’s genetic diversity corrected for sample size was calculated for independent populations using GenAlEx version 6.5 (53). The multilocus program (version 1.3b) was used to evaluate the presence and prevalence of linkage disequilibrium (LD) and phylogenetic compatibility as indicators of clonality and recombination (54). A diversity of samples and subsamples were analyzed, including those representing individual genetic clusters and geographic populations. The statistical significance of each test was determined using 1,000 permutations.
