# Supplementary material for: Global Population Genetic Analysis of Aspergillus fumigatus
Source: mSphere. 2017 Feb 1;2(1):e00019-17. doi: 10.1128/mSphere.00019-17 (PMC5288565; doi:10.1128/mSphere.00019-17)
Supplement: FIG S1 [file sph001172230sf1.pdf]

Results of Analysis of Molecular Variance

**Data Sheet**	File S1
**Data Title**	Geo separation
**No. Samples**	2026
**No. Pops**	13
**No. Permutations**	999
**N0**	115.699
**SSTOT**	7892.766

Percentages of Molecular Variance

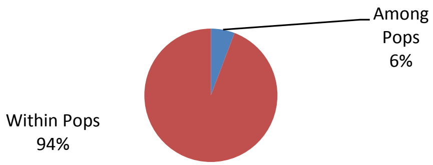

**Pop**	**Australia**	**Belgium**	**China**	**Cuba**	**France**	**Germany**	**India**	**Italy**	**Netherland**	**Norway**	**Spain**	**Switzerland**	**USA**
**n**	7	108	8	10	66	85	94	7	1081	203	186	70	101
**SSWP**	20.000	397.843	21.625	34.600	248.515	313.235	222.117	20.286	4188.110	708.246	705.323	269.671	379.564

Summary AMOVA Table

**Source**	**df**	**SS**	**MS**	**Est. Var.**	**%**
**Among Pops**	12	363.630	30.303	0.230	6%
**Within Pops**	2013	7529.136	3.740	3.740	94%
**Total**	2025	7892.766		3.970	100%

**Stat**	**Value**	**P(rand >= data) Probability, P(rand >= data), for PhiPT is based on standard permutation across the full data set.**	
**PhiPT**	0.058	0.001	**PhiPT = AP / (WP + AP) = AP / TOT**
**PhiPT max**	0.178		
**Phi'PT**	0.325		
**Nm (Haploid)**	8.146		Nm (Haploid) = [(1 / PhiPT) - 1] / 2

Key: AP = Est. Var. Among Pops, WP = Est. Var. Within Pops

Pairwise Population PhiPT Values

	**Australia**	**Belgium**	**China**	**Cuba**	**France**	**Germany**	**India**	**Italy**	**Netherland**	**Norway**	**Spain**	**Switzerland**	**USA**
**Australia**		0.038	0.001	0.032	0.008	0.015	0.001	0.050	0.005	0.001	0.025	0.010	0.011
**Belgium**	0.051		0.001	0.007	0.001	0.001	0.001	0.001	0.001	0.001	0.001	0.001	0.001
**China**	0.185	0.152		0.001	0.001	0.001	0.001	0.002	0.001	0.001	0.001	0.001	0.001
**Cuba**	0.068	0.049	0.163		0.030	0.021	0.001	0.113	0.027	0.001	0.019	0.024	0.014
**France**	0.075	0.035	0.115	0.035		0.001	0.001	0.009	0.001	0.001	0.001	0.003	0.001
**Germay**	0.061	0.058	0.137	0.034	0.049		0.001	0.028	0.001	0.001	0.001	0.001	0.001
**India**	0.357	0.225	0.375	0.297	0.183	0.273		0.001	0.001	0.001	0.001	0.001	0.001
**Italy**	0.113	0.103	0.208	0.048	0.088	0.044	0.373		0.001	0.001	0.004	0.001	0.003
**Netherland**	0.050	0.021	0.115	0.028	0.015	0.034	0.180	0.068		0.001	0.001	0.017	0.001
**Norway**	0.095	0.051	0.162	0.082	0.063	0.083	0.230	0.138	0.032		0.001	0.001	0.001
**Spain**	0.051	0.034	0.110	0.038	0.031	0.041	0.208	0.103	0.023	0.050		0.002	0.001
**Switzerland**	0.046	0.021	0.107	0.029	0.017	0.036	0.218	0.081	0.005	0.033	0.012		0.001
**USA**	0.068	0.060	0.120	0.044	0.053	0.054	0.257	0.105	0.044	0.074	0.037	0.035	

PhiPT Values below diagonal. Probability, P(rand >= data) based on 999 permutations is shown above diagonal.
