# Supplementary material for: Global Population Genetic Analysis of Aspergillus fumigatus
Source: mSphere. 2017 Feb 1;2(1):e00019-17. doi: 10.1128/mSphere.00019-17 (PMC5288565; doi:10.1128/mSphere.00019-17)
Supplement: FIG S2 [file sph001172230sf2.pdf]

Results of Analysis of Molecular Variance

**Data Sheet**	File S2
**Data Title**	Eco separation
**No. Samples**	1592
**No. Pops**	4
**No. Permutations**	999
**N0**	313.448
**SSTOT**	6187.791

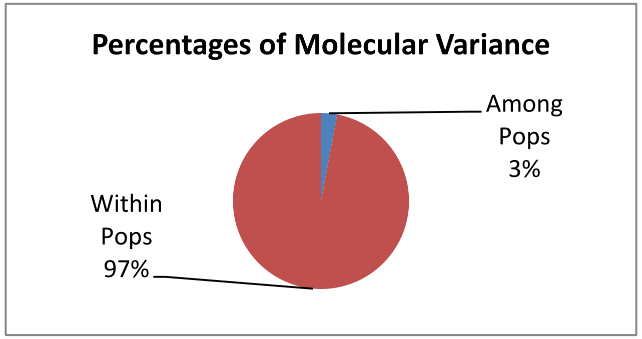

**Pop**	**Air**	**Clinical**	**Soil**	**Water**
**n**	529	858	117	88
**SSWP**	2060.386	3325.593	381.051	300.159

Summary AMOVA Table

**Source**	**df**	**SS**	**MS**	**Est. Var.**	**%**
**Among Pops**	3	120.602	40.201	0.116	3%
**Within Pops**	1588	6067.189	3.821	3.821	97%
**Total**	1591	6187.791		3.937	100%

**Stat**	**Value**	**P(rand >= data)**	
**PhiPT**	0.029	0.001	**PhiPT = AP / (WP + AP) = AP / TOT**
**PhiPT max**	0.158		
**Phi'PT**	0.187		
**Nm (Haploid)**	16.459		**Nm (Haploid) = [(1 / PhiPT) - 1] / 2**

Probability, P(rand >= data), for PhiPT is based on standard permutation across the full data set.

Key: AP = Est. Var. Among Pops, WP = Est. Var. Within Pops

Pairwise Population PhiPT Values

**Air**	**Clinical**	**Soil**	**Water**	
	0.001	0.001	0.001	**Air**
0.005		0.001	0.001	**Clinical**
0.089	0.082		0.001	**Soil**
0.032	0.034	0.115		**Water**

PhiPT Values below diagonal. Probability, P(rand >= data) based on 999 permutations is shown above diagonal.
