# Supplementary material for: Global Population Genetic Analysis of Aspergillus fumigatus
Source: mSphere. 2017 Feb 1;2(1):e00019-17. doi: 10.1128/mSphere.00019-17 (PMC5288565; doi:10.1128/mSphere.00019-17)
Supplement: FIG S3 [file sph001172230sf3.pdf]

**Data Sheet**	File S3
**Data Title**	**Mean allelic patterns across geographic and genetic populations**
**No. Loci**	9

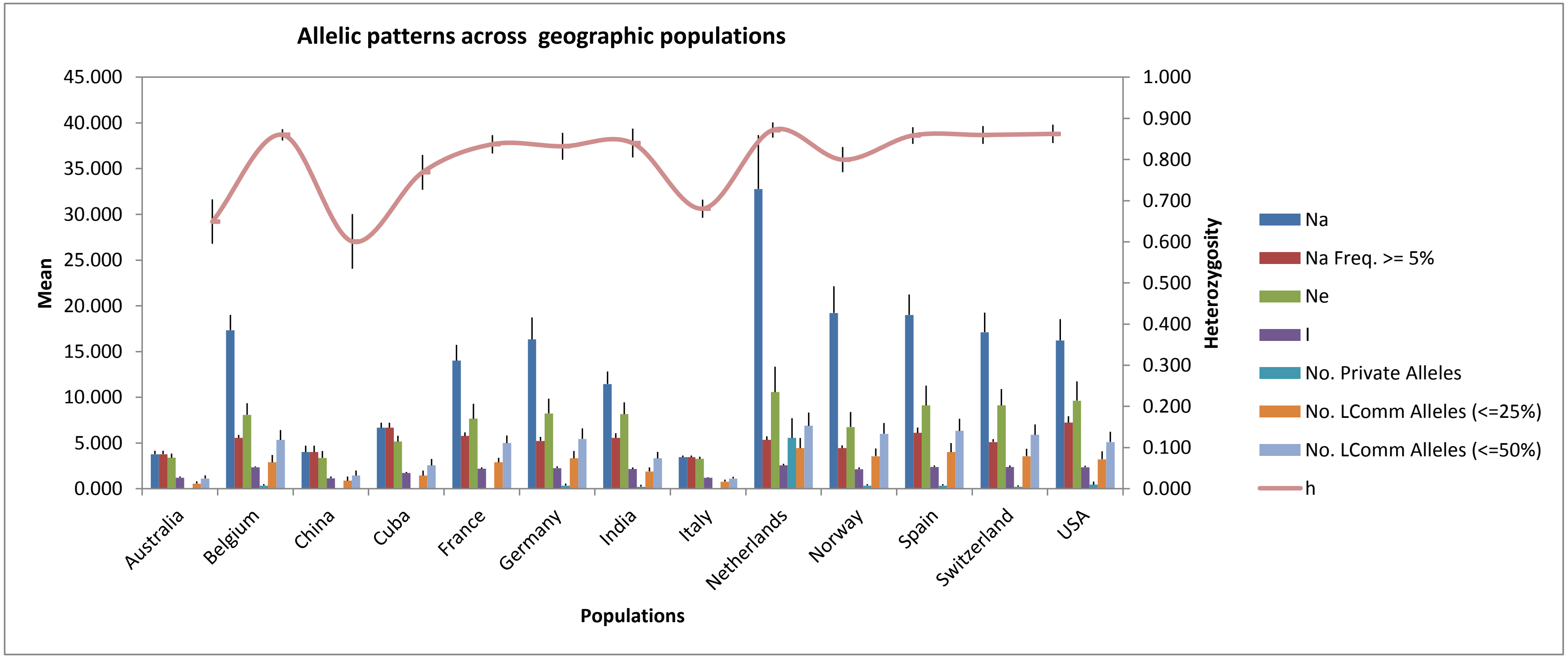

Mean values

**Population**	**Australia**	**Belgium**	**China**	**Cuba**	**France**	**Germany**	**India**	**Italy**	**Netherlands**	**Norway**	**Spain**	**Switzerland**	**USA**
**Na**	3.778	17.333	4.000	6.667	14.000	16.333	11.444	3.444	32.778	19.222	19.000	17.111	16.222
**Na Freq. >= 5%**	3.778	5.556	4.000	6.667	5.778	5.222	5.556	3.444	5.333	4.444	6.111	5.111	7.222
**Ne**	3.381	8.075	3.378	5.158	7.659	8.232	8.155	3.259	10.563	6.745	9.111	9.117	9.621
**I**	1.205	2.352	1.141	1.709	2.183	2.265	2.153	1.194	2.551	2.142	2.389	2.367	2.353
**No. Private Alleles**	0.000	0.333	0.000	0.000	0.000	0.333	0.222	0.000	5.556	0.333	0.333	0.222	0.444
**No. LComm Alleles (<=25%)**	0.556	2.889	0.889	1.444	2.889	3.333	1.889	0.778	4.444	3.556	4.000	3.556	3.222
**No. LComm Alleles (<=50%)**	1.111	5.333	1.444	2.556	5.000	5.444	3.333	1.111	6.889	6.000	6.333	5.889	5.111
**h**	0.649	0.860	0.601	0.769	0.837	0.832	0.840	0.681	0.872	0.800	0.858	0.860	0.862
**uh**	0.811	0.872	0.687	0.854	0.854	0.848	0.875	0.907	0.873	0.805	0.867	0.874	0.877

Standard Error (SE) values

Population	Australia	Belgium	China	Cuba	France	Germany	India	Italy	Netherlands	Norway	Spain	Switzerland	USA
**Na**	0.364	1.675	0.707	0.553	1.732	2.392	1.365	0.176	5.883	2.900	2.255	2.163	2.332
**Na Freq. >= 5%**	0.364	0.338	0.707	0.553	0.401	0.434	0.530	0.176	0.408	0.294	0.588	0.309	0.722
**Ne**	0.461	1.282	0.758	0.640	1.627	1.628	1.308	0.234	2.775	1.657	2.162	1.776	2.125
**I**	0.130	0.111	0.184	0.131	0.146	0.198	0.167	0.061	0.188	0.192	0.160	0.163	0.174
**No. Private Alleles**	0.000	0.167	0.000	0.000	0.000	0.236	0.222	0.000	2.167	0.167	0.167	0.147	0.338
**No. LComm Alleles (<=25%)**	0.242	0.790	0.455	0.556	0.484	0.799	0.455	0.222	1.107	0.852	1.000	0.801	0.862
**No. LComm Alleles (<=50%)**	0.351	1.093	0.530	0.689	0.816	1.156	0.707	0.200	1.457	1.179	1.333	1.136	1.124
**h**	0.054	0.014	0.066	0.042	0.022	0.033	0.035	0.022	0.019	0.031	0.020	0.022	0.022
**uh**	0.068	0.014	0.076	0.047	0.023	0.034	0.037	0.029	0.019	0.031	0.021	0.022	0.023

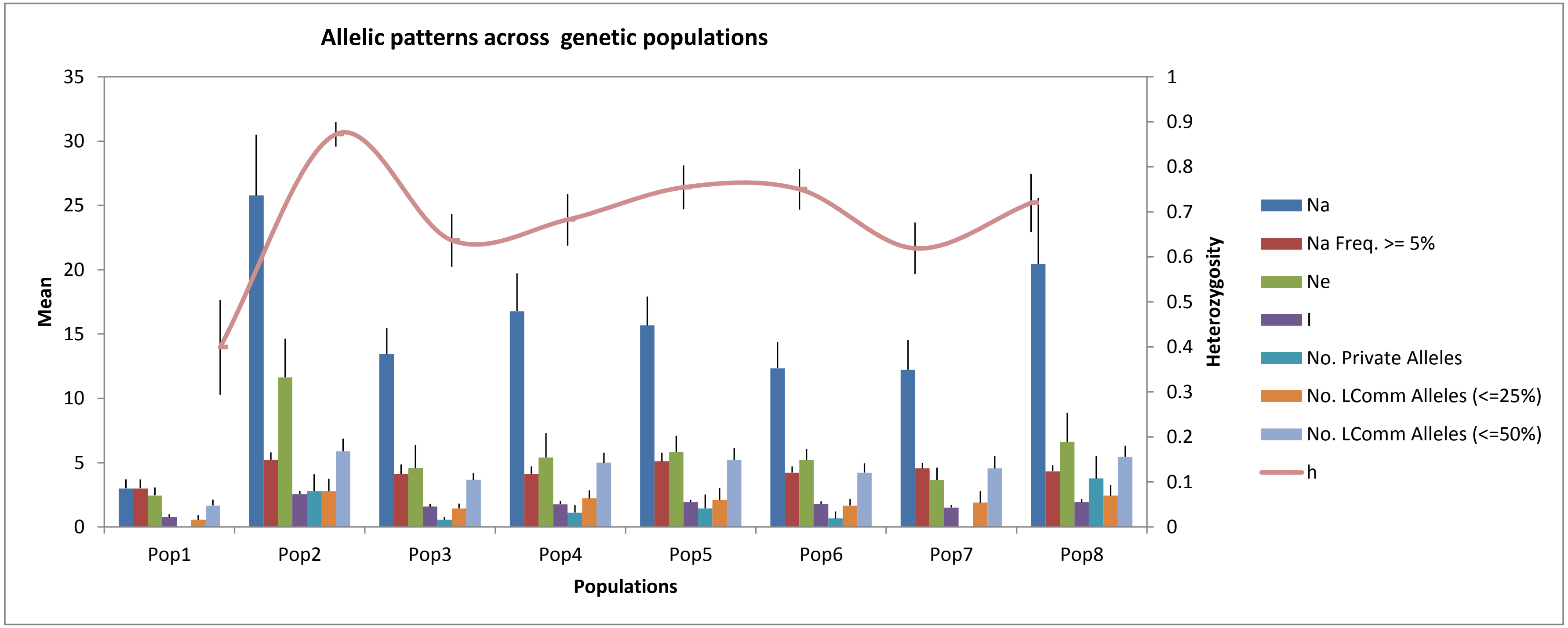

Mean values

Population	Pop1	Pop2	Pop3	Pop4	Pop5	Pop6	Pop7	Pop8
**Na**	3.000	25.778	13.444	16.778	15.667	12.333	12.222	20.444
**Na Freq. >= 5%**	3.000	5.222	4.111	4.111	5.111	4.222	4.556	4.333
**Ne**	2.445	11.634	4.579	5.399	5.828	5.202	3.650	6.616
**I**	0.756	2.558	1.581	1.770	1.924	1.796	1.515	1.909
**No. Private Alleles**	0.000	2.778	0.556	1.111	1.444	0.667	0.000	3.778
**No. LComm Alleles (<=25%)**	0.556	2.778	1.444	2.222	2.111	1.667	1.889	2.444
**No. LComm Alleles (<=50%)**	1.667	5.889	3.667	5.000	5.222	4.222	4.556	5.444
**h**	0.399	0.872	0.637	0.683	0.754	0.750	0.619	0.720
**uh**	0.456	0.876	0.644	0.687	0.758	0.759	0.624	0.722

Standard Error (SE) values

Population	Pop1	Pop2	Pop3	Pop4	Pop5	Pop6	Pop7	Pop8
**Na**	0.707	4.725	2.015	2.938	2.242	2.041	2.296	5.140
**Na Freq. >= 5%**	0.707	0.572	0.754	0.588	0.676	0.494	0.444	0.471
**Ne**	0.625	3.004	1.813	1.883	1.265	0.894	0.969	2.259
**I**	0.227	0.215	0.209	0.250	0.189	0.204	0.201	0.280
**No. Private Alleles**	0.000	1.310	0.242	0.588	1.082	0.553	0.000	1.762
**No. LComm Alleles (<=25%)**	0.377	0.969	0.377	0.619	0.920	0.527	0.904	0.835
**No. LComm Alleles (<=50%)**	0.471	0.992	0.527	0.782	0.940	0.722	0.973	0.884
**h**	0.105	0.027	0.059	0.058	0.049	0.045	0.057	0.065
**uh**	0.120	0.028	0.059	0.058	0.049	0.046	0.058	0.065
