# Supplementary material for: Global Population Genetic Analysis of Aspergillus fumigatus
Source: mSphere. 2017 Feb 1;2(1):e00019-17. doi: 10.1128/mSphere.00019-17 (PMC5288565; doi:10.1128/mSphere.00019-17)
Supplement: FIG S4 [file sph001172230sf4.pdf]

Results of Analysis of Molecular Variance

**Data Sheet**	File S4
**Data Title**	Gen Pop separation

**No. Samples**	1230
**No. Pops**	8
**No. Permutations**	999
**N0**	146.213
**SSTOT**	4830.342

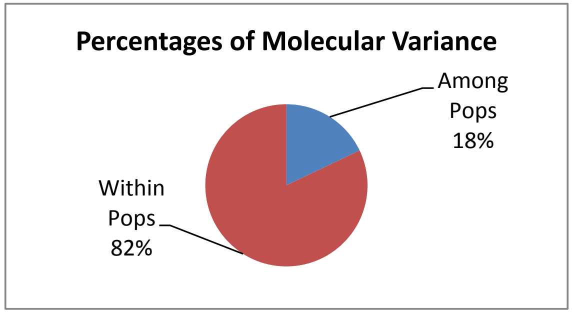

**Pop**	**Pop1**	**Pop2**	**Pop3**	**Pop4**	**Pop5**	**Pop6**	**Pop7**	**Pop8**
**n**	8	230	95	165	206	90	120	316
**SSWP**	14.375	906.652	273.526	507.527	699.500	303.944	334.200	1023.630

Summary AMOVA Table

**Source**	**df**	**SS**	**MS**	**Est. Var.**	**%**
**Among Pops**	7	766.987	109.570	0.727	18%
**Within Pops**	1222	4063.355	3.325	3.325	82%
**Total**	1229	4830.342		4.052	100%

**Stat**	**Value**	**P(rand >= data)**	
**PhiPT**	0.179	0.001	**PhiPT = AP / (WP + AP) = AP / TOT**
**PhiPT max**	0.263		
**Phi'PT**	0.683		
**Nm (Haploid)**	2.288		**Nm (Haploid) = [(1 / PhiPT) - 1] / 2**

Probability, P(rand >= data), for PhiPT is based on standard permutation across the full data set.

Key: AP = Est. Var. Among Pops, WP = Est. Var. Within Pops

Pairwise Population PhiPT Values

**Pop1**	**Pop2**	**Pop3**	**Pop4**	**Pop5**	**Pop6**	**Pop7**	**Pop8**	
	0.001	0.001	0.001	0.001	0.001	0.001	0.001	**Pop1**
0.216		0.001	0.001	0.001	0.001	0.001	0.001	**Pop2**
0.373	0.135		0.001	0.001	0.001	0.001	0.001	**Pop3**
0.344	0.099	0.194		0.001	0.001	0.001	0.001	**Pop4**
0.247	0.104	0.220	0.180		0.001	0.001	0.001	**Pop5**
0.300	0.083	0.195	0.157	0.162		0.001	0.001	**Pop6**
0.365	0.152	0.257	0.244	0.144	0.202		0.001	**Pop7**
0.336	0.145	0.242	0.249	0.184	0.188	0.243		**Pop8**

PhiPT Values below diagonal. Probability, P(rand >= data) based on 999 permutations is shown above diagonal.
