# Supplementary material for: Global Population Genetic Analysis of Aspergillus fumigatus
Source: mSphere. 2017 Feb 1;2(1):e00019-17. doi: 10.1128/mSphere.00019-17 (PMC5288565; doi:10.1128/mSphere.00019-17)
Supplement: FIG S5 [file sph001172230sf5.pdf]

File S5

Sample ID	Population	2A	2B	2C	3A	3B	3C	4A	4B	4C	No. Matches	Label
533	Clinical	10	14	10	17	13	14	7	5	5	0	A
1389	Env	10	14	10	17	13	14	7	5	5	2	A
738	Clinical	18	12	11	16	10	13	8	9	5	0	AA
186	Air	18	12	11	16	10	13	8	9	5	2	AA
957	Clinical	19	22	21	34	10	29	14	9	7	0	AAA
311	Air	19	22	21	34	10	29	14	9	7	2	AAA
1383	Clinical	25	19	19	26	19	17	10	16	8	0	AAAA
483	Air	25	19	19	26	19	17	10	16	8	2	AAAA
534	Clinical	10	14	10	17	13	14	7	5	6	0	B
10	Air	10	14	10	17	13	14	7	5	6	2	B
745	Clinical	18	12	11	26	11	20	8	8	5	0	BB
1460	Env	18	12	11	26	11	20	8	8	5	2	BB
994	Clinical	19	25	34	34	23	26	10	10	5	0	BBB
1519	Env	19	25	34	34	23	26	10	10	5	2	BBB
1268	Clinical	25	20	11	10	9	7	8	10	20	0	BBBB
492	Air	25	20	11	10	9	7	8	10	20	2	BBBB
536	Clinical	10	14	10	17	13	15	7	5	5	0	C
11	Air	10	14	10	17	13	15	7	5	5	2	C
750	Clinical	18	12	13	14	10	12	8	9	5	0	CC
1735	Water	18	12	13	14	10	12	8	9	5	2	CC
1021	Clinical	20	17	16	39	11	22	12	11	8	0	CCC
330	Air	20	17	16	39	11	22	12	11	8	2	CCC
1274	Clinical	25	20	13	37	9	10	8	10	8	0	CCCC
1575	Env	25	20	13	37	9	10	8	10	8	2	CCCC
538	Clinical	10	15	10	18	12	12	7	5	6	0	D
1592	Soil	10	15	10	18	12	12	7	5	6	2	D
756	Clinical	18	12	14	14	14	14	19	10	5	0	DD
197	Air	18	12	14	14	14	14	19	10	5	2	DD
1032	Clinical	20	19	15	39	12	7	10	11	12	0	DDD
333	Air	20	19	15	39	12	7	10	11	12	2	DDD
1256	Clinical	25	20	8	10	10	21	9	10	5	0	DDDD
1784	Water	25	20	8	10	10	21	9	10	5	2	DDDD
540	Clinical	10	16	10	17	13	19	7	5	6	0	E
20	Air	10	16	10	17	13	19	7	5	6	2	E
765	Clinical	18	12	14	27	11	14	9	11	7	0	EE
198	Air	18	12	14	27	11	14	9	11	7	2	EE
1035	Clinical	20	19	15	40	12	7	10	11	12	0	EEE
1529	Env	20	19	15	40	12	7	10	11	12	2	EEE
1257	Clinical	25	20	8	10	10	22	9	10	5	0	EEEE
486	Air	25	20	8	10	10	22	9	10	5	2	EEEE
542	Clinical	10	16	10	22	12	12	7	5	5	0	F
1595	Soil	10	16	10	22	12	12	7	5	5	2	F
784	Clinical	18	12	16	26	11	20	8	8	7	0	FF
206	Air	18	12	16	26	11	20	8	8	7	2	FF
1026	Clinical	20	19	8	31	14	20	9	9	5	0	FFF
1766	Water	20	19	8	31	14	20	9	9	5	2	FFF
1260	Clinical	25	20	9	10	10	7	8	10	10	0	FFFF
1785	Water	25	20	9	10	10	7	8	10	10	2	FFFF
545	Clinical	10	16	10	23	13	21	7	5	6	0	G
26	Air	10	16	10	23	13	21	7	5	6	2	G
785	Clinical	18	12	17	25	10	21	8	9	7	0	GG
1736	Water	18	12	17	25	10	21	8	9	7	2	GG
1061	Clinical	21	22	18	26	10	14	9	13	8	0	GGG
351	Air	21	22	18	26	10	14	9	13	8	2	GGG
1275	Clinical	25	21	8	29	9	6	8	10	20	0	GGGG
494	Air	25	21	8	29	9	6	8	10	20	2	GGGG
547	Clinical	10	16	10	24	11	8	7	5	5	0	H
30	Air	10	16	10	24	11	8	7	5	5	2	H
706	Clinical	18	12	8	27	10	19	9	9	5	0	HH
1730	Water	18	12	8	27	10	19	9	9	5	2	HH
1063	Clinical	21	22	18	26	10	15	9	13	8	0	HHH
1681	Soil	21	22	18	26	10	15	9	13	8	2	HHH
1295	Clinical	25	22	19	28	9	26	10	9	5	0	HHHH
1577	Env	25	22	19	28	9	26	10	9	5	2	HHHH
549	Clinical	10	16	10	25	11	8	7	5	6	0	I
1597	Soil	10	16	10	25	11	8	7	5	6	2	I
710	Clinical	18	12	8	27	10	20	9	9	5	0	II
1669	Soil	18	12	8	27	10	20	9	9	5	2	II
1070	Clinical	21	25	18	27	12	7	20.3	10	8	0	III
1682	Soil	21	25	18	27	12	7	20.3	10	8	2	III
1297	Clinical	25	22	20	27	9	26	10	9	5	0	IIII
508	Air	25	22	20	27	9	26	10	9	5	2	IIII
551	Clinical	10	16	10	25	12	8	7	5	6	0	J
33	Air	10	16	10	25	12	8	7	5	6	2	J
720	Clinical	18	12	8	28	10	20	9	9	5	0	JJ
1732	Water	18	12	8	28	10	20	9	9	5	2	JJ
1077	Clinical	21	25	19	27	12	7	20.3	10	8	0	JJJ
361	Air	21	25	19	27	12	7	20.3	10	8	2	JJJ
1300	Clinical	25	26	9	10	10	7	9	9	5	0	JJJJ
510	Air	25	26	9	10	10	7	9	9	5	2	JJJJ
554	Clinical	10	16	10	26	12	8	7	5	6	0	K
42	Air	10	16	10	26	12	8	7	5	6	2	K
721	Clinical	18	12	8	28	10	21	9	9	5	0	KK
170	Air	18	12	8	28	10	21	9	9	5	2	KK
1080	Clinical	21	25	19	27	12	7	21	10	8	0	KKK
363	Air	21	25	19	27	12	7	21	10	8	2	KKK
1313	Clinical	26	20	8	25	9	7	8	10	10	0	KKKK
517	Air	26	20	8	25	9	7	8	10	10	2	KKKK
556	Clinical	10	16	10	30	13	21	7	5	5	0	L
43	Air	10	16	10	30	13	21	7	5	5	2	L

730	Clinical	18	12	8	31	10	20	9	9	5	0	LL
178	Air	18	12	8	31	10	20	9	9	5	2	LL
1089	Clinical	22	19	15	37	11	21	10	26	5	0	LLL
369	Air	22	19	15	37	11	21	10	26	5	2	LLL
1317	Clinical	26	20	8	35	9	7	8	10	20	0	LLLL
518	Air	26	20	8	35	9	7	8	10	20	2	LLLL
557	Clinical	10	16	10	31	13	21	7	5	5	0	M
44	Air	10	16	10	31	13	21	7	5	5	2	M
816	Clinical	18	17	16	36	21	24	15.3	9	8	0	MM
1474	Env	18	17	16	36	21	24	15.3	9	8	2	MM
1101	Clinical	23	16	8	56	10	20	12.3	10	8	0	MMM
375	Air	23	16	8	56	10	20	12.3	10	8	2	MMM
575	Clinical	11	12	17	25	22	21	14	8	5	0	N
1706	Water	11	12	17	25	22	21	14	8	5	2	N
836	Clinical	18	19	11	27	10	34	20	11	5	0	NN
1748	Water	18	19	11	27	10	34	20	11	5	2	NN
1117	Clinical	23	19	15	47	11	7	13	9	5	0	NNN
388	Air	23	19	15	47	11	7	13	9	5	2	NNN
588	Clinical	11	14	14	27	11	5	15	8	7	0	O
1407	Env	11	14	14	27	11	5	15	8	7	2	O
843	Clinical	18	19	11	27	10	35	20	11	5	0	OO
244	Air	18	19	11	27	10	35	20	11	5	2	OO
1125	Clinical	23	19	15	48	11	7	13	9	5	0	OOO
394	Air	23	19	15	48	11	7	13	9	5	2	OOO
593	Clinical	13	10	9	10	11	9	8	9	19	0	P
1410	Env	13	10	9	10	11	9	8	9	19	2	P
819	Clinical	18	19	8	10	10	20	9	9	10	0	PP
224	Air	18	19	8	10	10	20	9	9	10	2	PP
1130	Clinical	23	19	15	49	11	7	13	9	5	0	PPP
1548	Env	23	19	15	49	11	7	13	9	5	2	PPP
600	Clinical	13	19	8	22	10	10	9	10	21	0	Q
1708	Water	13	19	8	22	10	10	9	10	21	2	Q
824	Clinical	18	19	8	25	23	20	15	9	5	0	QQ
228	Air	18	19	8	25	23	20	15	9	5	2	QQ
1136	Clinical	23	19	15	50	11	7	13	9	5	0	QQQ
405	Air	23	19	15	50	11	7	13	9	5	2	QQQ
622	Clinical	14	12	9	26	10	20	8	8	10	0	R
1417	Env	14	12	9	26	10	20	8	8	10	2	R
830	Clinical	18	19	8	28	14	21	9	9	5	0	RR
1742	Water	18	19	8	28	14	21	9	9	5	2	RR
1150	Clinical	23	19	16	29	17	7	10	26	5	0	RRR
1549	Env	23	19	16	29	17	7	10	26	5	2	RRR
646	Clinical	14	20	11	34	9	7	8	10	12	0	S
1427	Env	14	20	11	34	9	7	8	10	12	2	S
853	Clinical	18	20	9	10	10	12	8	10	5	0	SS
1749	Water	18	20	9	10	10	12	8	10	5	2	SS
1153	Clinical	23	19	16	30	17	7	10	26	5	0	SSS
411	Air	23	19	16	30	17	7	10	26	5	2	SSS
640	Clinical	14	20	8	36	13	10	8	10	5	0	T
118	Air	14	20	8	36	13	10	8	10	5	2	T
872	Clinical	18	21	19	22	11	31	10	8	8	0	TT
1751	Water	18	21	19	22	11	31	10	8	8	2	TT
1154	Clinical	23	19	16	44	20	7	13.3	9	7	0	TTT
415	Air	23	19	16	44	20	7	13.3	9	7	2	TTT
1350	Clinical	14	20	9	31	9	10	8	10	28	0	U
1658	Soil	14	20	9	31	9	10	8	10	28	2	U
876	Clinical	18	21	27	62	11	30	17.3	9	5	0	UU
1498	Env	18	21	27	62	11	30	17.3	9	5	2	UU
1177	Clinical	23	21	8	48	9	6	8	9	5	0	UUU
433	Air	23	21	8	48	9	6	8	9	5	2	UUU
666	Clinical	15	19	8	26	10	7	8	9	5	0	V
1720	Water	15	19	8	26	10	7	8	9	5	2	V
924	Clinical	18	25	19	28	11	7	17.3	10	8	0	VV
1511	Env	18	25	19	28	11	7	17.3	10	8	2	VV
1199	Clinical	23	23	15	38	11	49	10	26	8	0	VVV
444	Air	23	23	15	38	11	49	10	26	8	2	VVV
667	Clinical	15	19	8	33	15	20	9	10	5	0	W
136	Air	15	19	8	33	15	20	9	10	5	2	W
932	Clinical	18	28	8	16	10	18	8	9	5	0	WW
1755	Water	18	28	8	16	10	18	8	9	5	2	WW
1216	Clinical	23	24	15	48	13	7	10	9	5	0	WWW
1691	Soil	23	24	15	48	13	7	10	9	5	2	WWW
668	Clinical	15	19	8	34	15	20	9	10	5	0	X
1722	Water	15	19	8	34	15	20	9	10	5	2	X
943	Clinical	19	12	12	25	25	35	12	12	5	0	XX
295	Air	19	12	12	25	25	35	12	12	5	2	XX
1381	Clinical	25	10	8	86	9	12	8	7	5	0	XXX
1695	Soil	25	10	8	86	9	12	8	7	5	2	XXX
670	Clinical	15	19	9	10	10	13	9	10	5	0	Y
1723	Water	15	19	9	10	10	13	9	10	5	2	Y
944	Clinical	19	12	15	17	25	12	16	10	5	0	YY
297	Air	19	12	15	17	25	12	16	10	5	2	YY
1242	Clinical	25	16	23	22	11	20	12	11	8	0	YYY
476	Air	25	16	23	22	11	20	12	11	8	2	YYY
687	Clinical	17	12	13	16	10	22	8	8	8	0	Z
147	Air	17	12	13	16	10	22	8	8	8	2	Z
956	Clinical	19	22	21	33	10	29	14	9	7	0	ZZ
310	Air	19	22	21	33	10	29	14	9	7	2	ZZ
1250	Clinical	25	19	16	26	13	7	10	16	8	0	ZZZ
1700	Soil	25	19	16	26	13	7	10	16	8	2	ZZZ
