# Supplementary material for: Global Population Genetic Analysis of Aspergillus fumigatus
Source: mSphere. 2017 Feb 1;2(1):e00019-17. doi: 10.1128/mSphere.00019-17 (PMC5288565; doi:10.1128/mSphere.00019-17)
Supplement: FIG S6 [file sph001172230sf6.pdf]

Results of Analysis of Molecular Variance

**Data Sheet**	File S6
**Data Title**	Res seperation

**No. Samples**	102
**No. Pops**	3
**No. Permutations**	999
**N0**	27.108
**SSTOT**	219.902

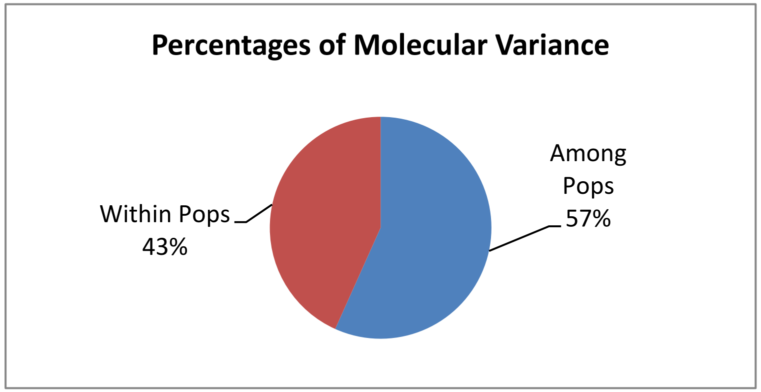

**Pop**	**China**	**India**	**Netherlands**
**n**	8	61	33
**SSWP**	21.625	0.000	104.909

Summary AMOVA Table

**Source**	**df**	**SS**	**MS**	**Est. Var.**	**%**
**Among Pops**	2	93.368	46.684	1.675	57%
**Within Pops**	99	126.534	1.278	1.278	43%
**Total**	101	219.902		2.953	100%

**Stat**	**Value**	**P(rand >= data)**	
**PhiPT**	0.567	0.001	**PhiPT = AP / (WP + AP) = AP / TOT**
**PhiPT max**	0.685		
**Phi'PT**	0.828		
**Nm (Haploid)**	0.382		**Nm (Haploid) = [(1 / PhiPT) - 1] / 2**

Probability, P(rand >= data), for PhiPT is based on standard permutation across the full data set.

Key: AP = Est. Var. Among Pops, WP = Est. Var. Within Pops

**China**	**India**	**Netherlands**	
	0.001	0.001	**China**
0.897		0.001	**India**
0.232	0.566		**Netherlands**

PhiPT Values below diagonal. Probability, P(rand >= data) based on 999 permutations is shown above diagonal.
